# Photocatalytic Detoxification of Some Insecticides in Aqueous Media Using TiO_2_ Nanocatalyst

**DOI:** 10.3390/ijerph18179278

**Published:** 2021-09-02

**Authors:** Ahmed Massoud, Aly Derbalah, Ibrahim El-Mehasseb, Moustafa Saad Allah, Mohamed S. Ahmed, Ashraf Albrakati, Ehab Kotb Elmahallawy

**Affiliations:** 1 Pesticides Chemistry and Toxicology Department, Faculty of Agriculture, Kafrelsheikh University, Kafrelsheikh 33516, Egypt; ahmed.masoud@agr.kfs.edu.eg (A.M.); ali.derbala@agr.kfs.edu.eg (A.D.); mostafa.allah@agr.kfs.edu.eg (M.S.A.); 2 Chemistry Department, Faculty of Science, Kafrelsheikh University, Kafrelsheikh 33516, Egypt; ibrahim.elmehasseb@sci.kfs.edu.eg; 3 Department of Pathology, Faculty of Veterinary Medicine, Kafrelsheikh University, Kafrelsheikh 33516, Egypt; mohamed.abdelrahman1@vet.kfs.edu.eg; 4 Department of Human Anatomy, College of Medicine, Taif University, P.O. Box 11099, Taif 21944, Saudi Arabia; a.albrakati@tu.edu.sa; 5 Department of Biomedical Sciences, University of Leon, 24004 Leon, Spain; 6 Department of Zoonoses, Faculty of Veterinary Medicine, Sohag University, Sohag 82524, Egypt

**Keywords:** photocatalytic, detoxification insecticides, aqueous media, TiO_2_ nanocatalyst, nanocatalyst, toxicity, histopathology

## Abstract

The present study was performed to fabricate a titanium dioxide (TiO_2_) nanocatalyst with proper characteristics for the removal of some insecticides (dimethoate and methomyl) from aqueous media. A TiO_2_ catalyst of regular (TiO_2_—commercial—/H_2_O_2_/UV) or nano (TiO_2_—synthesized—/H_2_O_2_/UV) size was employed as an advanced oxidation process by combining it with H_2_O_2_ under light. Moreover, the total detoxification of insecticides after treatment with the most effective system (TiO_2_(s)/H_2_O_2_/UV) was also investigated through exploring the biochemical alterations and histopathological changes in the liver and kidneys of the treated rats. Interestingly, the present study reported that degradation rates of the examined insecticides were faster using the TiO_2_ catalyst of nano size. Complete degradation of the tested insecticides (100%) was achieved under the TiO_2_(s)/H_2_O_2_/UV system after 320 min of irradiation. The half-life values of the tested insecticides under H_2_O_2_/TiO_2_(c)/UV were 43.86 and 36.28 for dimethoate and methomyl, respectively, whereas under the H_2_O_2_/TiO_2_(c)/UV system, the half-life values were 27.72 and 19.52 min for dimethoate and methomyl, respectively. On the other hand, no significant changes were observed in the biochemical and histopathological parameters of rats administrated with water treated with TiO_2_(s)/H_2_O_2_/UV compared to the control, indicating low toxicity of the TiO_2_ nanocatalyst-. Altogether, the advanced oxidation processes using TiO_2_ nanocatalyst can be considered as a promising and effective remediation technology for the complete detoxification of methomyl and dimethoate in water. However, further future research is needed to identify the possible breakdown products and to verify the safety of the used nanomaterials.

## 1. Introduction

Water is a prerequisite for life, a key reason for human existence, and it is abundant on earth. However, approximately 97% of the earth’s water is saltwater and the remaining 2.5% is freshwater, and only less than 1% of the world’s freshwater resources are readily available for human use [1]. Importantly, nearly 2.2 million annual deaths in developing countries, particularly children, result from diseases associated with a lack of safe drinking water in adequate sanitation and poor hygienic practices [2,3].

It should be stressed that pesticide contamination of surface and groundwater from agricultural use has been a concern for a long time [4,5,6,7,8,9]. In the last few decades, contamination by organochlorine pesticides (OCPs), organophosphorus, and carbamates has attracted considerable attention due to their resulting toxicity and persistence in the environment. Among others, Methomyl is highly soluble in water, but it is of low sorption affinity in soils, resulting in easier contamination of surface and groundwater resources [10]. On the other hand, Dimethoate is a commonly used organophosphorus insecticide in agricultural activities, including in the Mediterranean region [11,12]. Inside the body, Dimethoate converts to the active metabolite, demithoxon, which cumulatively represents a major risk of adverse health effects [13,14]. Collectively, the random use of pesticides results in the accumulation of a large amount of these toxic compounds and their residues in the food chain, drinking water, and the environment, which contributes to threatening ecosystems and human health [15].

Several conventional methods have been adopted for treatment of surface and groundwater sources contaminated with pesticides; however, the advanced oxidation method has been considered among the most effective technologies [16,17,18,19]. Advanced oxidation processes (AOPs), constituted by the combination of several oxidants, are characterized by the generation of very reactive and oxidizing free radicals in aqueous solutions such as hydroxyl radicals, which possess great destruction power [5,6,7,16,17,18]. To the best of our knowledge, conventional photocatalysts such as TiO_2_ and ZnO can be used in low initial concentrations not only to remove pesticides [5,6,7,9,17,20,21], but also other organic pollutants [22]. Heterogeneous photocatalysis containing semiconducting photocatalysts used in AOPs has recently emerged as an advanced oxidation process in an environmental decontamination method suitable for the treatment of water, aqueous wastes, and wastewater [17,23,24,25]. Nanostructured semiconductors such as TiO_2_ can be considered a potential candidate for the mineralization of toxic organic compounds, hazardous inorganic constituents [9,17,26], and bacteria disinfection [27], owing to their strong oxidizing ability, i.e., hydroxyl radical (OH^·^) relative to the bulk one.

However, after remediation of pesticide residues in water, a toxicity assessment is still required to confirm the complete detoxification of any possible potential hazard from both original pollutants and their metabolites [6,7,16]. This method remains inaccurate because sometimes the degradation products of pesticides are more toxic than the pesticides themselves. Interestingly, toxicity testing has been widely used to determine the potential toxicity of a certain compound or sample of water to biological organisms combined with measurement of their extent. Unlike chemical analysis, toxicity testing targets the detection of the toxic compounds on the basis of their biological activity without the necessity of prior knowledge about the toxicant, and therefore toxicity testing offers many advantages over chemical analysis [28].

In this context, the present study aims to evaluate the efficacy of advanced processes using TiO_2_ in regular and nano sizes for the complete degradation of the tested insecticides (dimethoate and methomyl) in aqueous media. This study also intends to confirm the complete detoxification of insecticide-contaminated water after remediation with the most effective treatment (TiO_2_(s)/H_2_O_2_/UV) regarding the alteration of several biochemical parameters and reporting histopathological changes in the liver and kidneys of the treated rats.

## 2. Material and Methods

### 2.1. Material

#### 2.1.1. Chemicals

Dimethoate and methomyl with 99.5% purity were obtained from the Central Laboratory for Pesticides, Agriculture Research Centre, Cairo, Egypt. Titanium dioxide anatase (TiO_2_) with 99% purity was also obtained from Winlab Company for Laboratory Fine Chemicals, Kolkata, India. A hydrogen peroxide solution (H_2_O_2_) with 50% (*w*/*v*) purity was obtained from Piochem for Laboratory Chemicals Company, Cairo, Egypt. Titanium tetrachloride (TiCl_4_) with 98% purity was obtained from BHD Limited Pode Company, England, Bridgend, UK. Ethanol (C_2_H_5_OH) with 95% purity was obtained from Piochem for Laboratory Chemicals Company, Cairo, Egypt.

#### 2.1.2. Synthesis of TiO_2_ Nanoparticles

A nanostructured TiO_2_ catalyst was prepared by hydrolysis of titanium isopropoxide or titanium tetrachloride [29]. Briefly, the typical synthesis involved dropping 4 mL TiCl_4_ solution into 400 mL of a mixture of ethanol and distilled water (4:1). The mixture was then refluxed at 80 °C under stirring; followed by the formation of a white suspension of TiO_2_ nanoparticles after approximately 120 min of reflux. The process continued for 120 min after the formation of the nanoparticles to eliminate the majority of the chloride ions from the suspension as Hydrogen chloride (HCl) gas. The nanoparticles were thrown down by centrifugation at 6000 rpm for 30 min. Subsequently, the precipitate was filtered and washed in water several times until the precipitate was free from impurities, then dried in a drying oven at 50 °C and calcined at 400 °C for 4 h to obtain a white powder of TiO_2_ (NPs) [30]. Physical characterizations of the synthesized and commercial TiO_2_ were performed using a scanning electron microscopy (SEM; JEOL Ltd.—Tokyo, Japan; JSM 6510) and transmission electron microscopy (TEM, JEOL JEM-2100). A Rigaku-D/max 2500 diffractometer (Rigaku, Shibuya-ku, Japan) with Cu-Kα radiation (λ = 0.15418 nm) was used to measure X-ray diffraction (XRD) for crystallization identification solid diffuse reflectance. The UV-Vis spectroscopy technique was employed to characterize the electronic structure of TiO_2_ nanoparticles.

#### 2.1.3. Photochemical Remediation of the Tested Insecticides

This experiment was designed to assess the efficiency of the advanced oxidation method using TiO_2_ in nanoparticles (synthesized) and regular sized (commercial) in the disposal of insecticides in water. A ultraviolet (UV) mercury lamp (model VL-4 LC—80 W, Ettlingen, Germany; with a wavelength range of 254 to 365 nm) was used for the irradiation of the tested insecticides (methomyl and dimethoate) in the aqueous solution. The solution was prepared by adding the desired amount (5 ppm) of insecticides separately in distilled water, followed by careful agitation. The freshly prepared TiO_2_ (synthesized or commercial) (300 mg/L) was added to the solution and shacked carefully before irradiation, followed by the addition of 20 mg/L H_2_O_2_. After that, distilled water was added to reach the total volume of 100 mL. Then the pH was adjusted to 7 with NaOH as the optimum pH for the TiO_2_ catalyst (pH meter Jenway—Staffordshire, UK, Model 3510, PH/mV/Temperature Meter) [16]. The suspension was maintained in the dark for 30 min before irradiation to reach the maximum adsorption of the pesticide onto the semiconductor surface. The distance between the lamp and the solution containing the insecticides was 19 cm and the wavelength used was 365 nm [31]. After starting the irradiation, the solutions from the irradiated samples were removed at regular intervals (0, 10, 20, 40, 60, 80, 160, 320, and 360 min) for high-performance liquid chromatography (HPLC) analysis [19]. A blank experiment was performed as mentioned, but in the absence of light to assess the abiotic loss of insecticides. All experiments were repeated three times and the average values are reported. The samples were then filtered using a Millipore syringe filter of 0.45 μm for HPLC analysis.

#### 2.1.4. HPLC Analysis

The irradiated samples were analyzed directly for methomyl and dimethoate using HPLC, according to the methods described in the literature [21,32]. For methomyl analysis, a mixture of acetonitrile and distilled water (20:80) was used as the mobile phase; an isocratic elution mode (i.e., in which the composition of the mobile phase is kept constant) was used. The flow rate of the mobile phase was maintained at 0.7 mL min^−1^, and the column type used was C8 zorbax (250 mm × 4.6 mm × 5 μm). A UV detector with a wavelength of 231 nm was used at a retention time of 3.84 min [21]. For dimethoate analysis, a mixture of acetonitrile and distilled water (60:40) was used as the mobile phase; an isocratic elution mode was used. The flow rate of the mobile phase was maintained at 1 mL min^−1^, and the column (inner diameter, 4.6 mm; length, 250 mm) was filled with Wakosil-II 5 C18-100 (Wako Pure Chemicals, Ltd., Osaka, Japan). A UV detector with a wavelength of 210 nm was used, and the retention time was 3.30 min [32].

#### 2.1.5. Degradation Kinetics

The degradation efficiency (%) was calculated as follows Equation (1):

Efficiency (%) = [(C_0_ − C)/C_0_] × 100 (1)

where C_0_ is the initial insecticide concentration and C is the insecticide concentration after photoirradiation [33]. To determine the degradation rate, plots of the Ln concentration against time were obtained. The degradation rate constant (slope) k was calculated from the first-order Equation (2):

C_t_ = C_0_e^−kt^ (2)

where C_t_ represents the insecticide concentration at the time t, C_0_ represents the initial concentration, and k is the degradation rate constant. When the concentration falls to 50% of its initial concentration, the half-life (t_1/2_) was estimated as shown in Equation (3) [34].

t_1/2_ = 0.693/K (3)

### 2.2. Toxicity Test

#### 2.2.1. Ethical Statement

Ethical approval was performed according to the ethical standards of Veterinary Medicine, Kafrelsheikh University, Egypt, which complies with all relevant Egyptian legislations. The ethical approval code number of the study is KFS-2019/10.

#### 2.2.2. Animals

Adult Sprague–Dawley (SD) rats of 100–120 g were obtained from the faculty of Veterinary Medicine, Kafrelsheikh University, and were acclimatized for one week before the experiment. All rats were housed in polypropylene cages, under standard conditions of 22 ± 2 °C temperature, 30–70% relative humidity, and a 12 h light/dark cycle. The cages were well ventilated, and the standard rat feed and water were provided ad libitum [35].

#### 2.2.3. Animal Treatment

The toxicity protocol used in this study was as follows: 1 mL of freshly prepared water containing dimethoate or methomyl, after remediation with (TiO_2_(s)/H_2_O_2_/UV) for six hours, was administered once as an oral dose to each rat. The dose of the used insecticides given to the treated rats, if no degradation induced, was equal to 500 µg/kg body weight (b.w). Rats were administrated insecticides using an injection syringe with a ball in the front to prevent the occurrence of bleeding or injury to the animal. The animals were divided into four groups (6 rats per each). Two groups were orally administrated one time with 1 mL of water contaminated with methomyl and dimethoate after remediation (6 h) with TiO_2_(nano)/H_2_O_2_/UV. The third group was orally administrated one time with 1 mL water containing TiO_2_(s)/H_2_O_2_ without insecticides. The fourth group of rats was treated with pure water only and considered as the control. After 28 days of oral administration, biochemical and histopathological tests were carried out to confirm the complete detoxification of insecticides in the treated water and to assess the toxicity of TiO_2_(s)/H_2_O_2_ without the tested insecticides relative to control. The animals were also observed for clinical signs of toxicity (macroscopic investigation) once a day throughout the entire observation period and the effects were observed in all six animals. All animal studies were approved by our Institutional Animal Ethics Committee.

#### 2.2.4. Biochemical Assays

A total of 6 blood samples were taken from each treatment group (1 sample per animal). Blood samples were collected and centrifuged at 4500 rpm for 20 min, and then serum was obtained for the determination of the enzyme’s activities. The colorimetric methods of Waber (1966) [36], Schirmeistar (1964) [37], Reitman and Frankel (1957) [38], and Habig and Jakoby (1974) [39] were used to determine the level of acetylcholinesterase, glutamic-pyruvic transaminase (GPT), glutamic-oxaloacetic transaminase (GOT), and glutathione-s-transferase (GST), respectively. The determination of enzymes in the serum of rats after treatment, except for the GST enzyme, was performed using a UV/VIS spectrometer.

#### 2.2.5. Histopathological Examination

All rats were anesthetized and sacrificed before performing the postmortem examination, and all lesions were recorded. Specimens from the liver and kidneys (one specimen from each organ per animal) were collected and maintained in neutral buffered formalin 10% for histopathological examination. The specimens were then dehydrated in ascending grades of alcohols, cleared in xylene, embedded in paraffin wax, sectioned at 4 µm, stained with hematoxylin and eosin (HE) stains, and examined by light microscopy [40].

### 2.3. Statistical Analysis

Enzyme activity data were statistically analyzed using one-way analysis of variance (ANOVA). The Duncan multiple ranges test was used to separate means using the SPSS program (statistical software package for windows Version 11.0) [41]. *p* ≤ 0.05 was set as the limit of significance.

## 3. Results

### 3.1. Characterization of the Catalyst

Figure S1 displays the SEM images of the synthesized and commercial TiO_2_, respectively. The synthesized TiO_2_ is spherically shaped with a diameter ranging from 30 to 40 nm (Figure S1a), while the commercial TiO_2_ is also spherical in shape but with a large diameter, ranging from 124 to 206 nm (Figure S1b). Additional investigations on the structures of the synthesized and commercial TiO_2_ were performed using TEM. Figure S2a shows the TEM image of the synthesized TiO_2_; the shape of the majority of these particles is spherical with only a small quantity of hexagonal diameters. The particle size ranged from 8.52 to 34.56 nm. Figure S2b shows the shape of the commercial TiO_2_, which is spherical with a small quantity of hexagonal diameters, ranging from 33.33 to 100 nm and with a length of 100–273.33 nm.

The solid diffuse reflectance UV-Vis spectroscopy technique was employed to characterize the electronic structure of TiO_2_ nanoparticles. Figure S3a,b shows the absorption edges at 388 nm for the synthesized TiO_2_ and at 392 nm for the commercial TiO_2_. The band gaps were 3.19 and 3.16 eV for the synthesized and commercial TiO_2_, respectively, which are in agreement with their surface areas. The small particle size of the synthesized TiO_2_ has a high surface area, and consequently, a high activity for organic pollutants degradation [42,43]. The X-ray diffraction pattern of the synthesized and commercial TiO_2_ are shown in Figure S4a,b. The 2θ values of the synthesized TiO_2_-NPs are observed at 25.32, 27.25, 47.57, 48.25, 54.29, and 62.5°, and are mostly an anatase phase with a small amount of a rutile phase represented by the characteristic peak at 27.25°. For the commercial TiO_2_, the peaks are located at 25.06, 37, 57, 47, 83, 54.86, and 62.50°, which are similar to the reported values for the pure anatase TiO_2_ phase [44]. Additionally, the strong diffraction peaks at approximately 25° (101) and 48° (200) are attributed to TiO_2_ in the anatase phase [45]. The absence of spurious diffraction peaks is a good indication of crystallographic purity [46]. Generally, the crystallite size is considered the main factor producing the intense and broad peak compared to the smaller size, which gives broader and less intense peaks. The average crystal sizes (d) of the synthesized and commercial TiO_2_ were calculated based on the width of the peak attributed to (101) planes by applying Scherrer’s formula [47]. The particle sizes of both synthesized and commercial TiO_2_ according to Scherrer’s formula are 11.4 and 65.3 nm, respectively.

### 3.2. Photocatalytic Degradation of the Tested Insecticides in Aqueous Media

**Figure 1:**
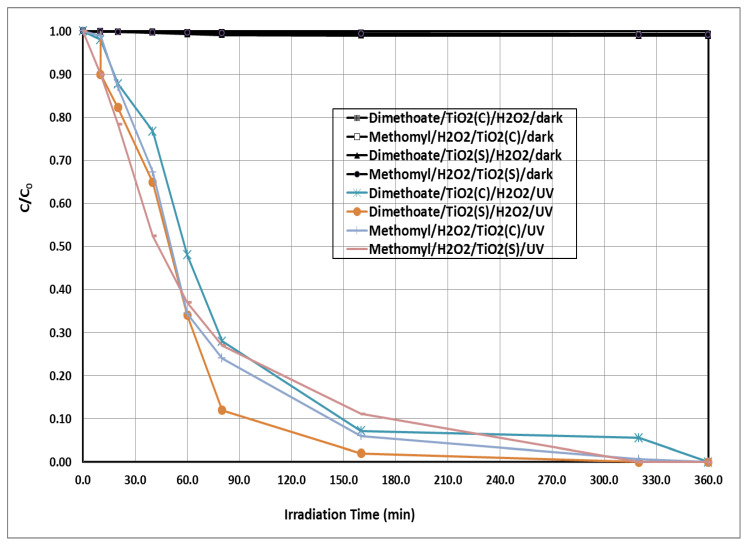
Photochemical degradation of the tested insecticides at initial concentration of 5 ppm using TiO_2_(c)/H_2_O_2_/UV and TiO_2_(s)/H_2_O_2_/UV. H_2_O_2_ = 20 mg/L, TiO_2_ = 300 mg/L, pH = 7. C = concentration at t time, C_0_ = initial concentration at zero time.

**Figure 2:**
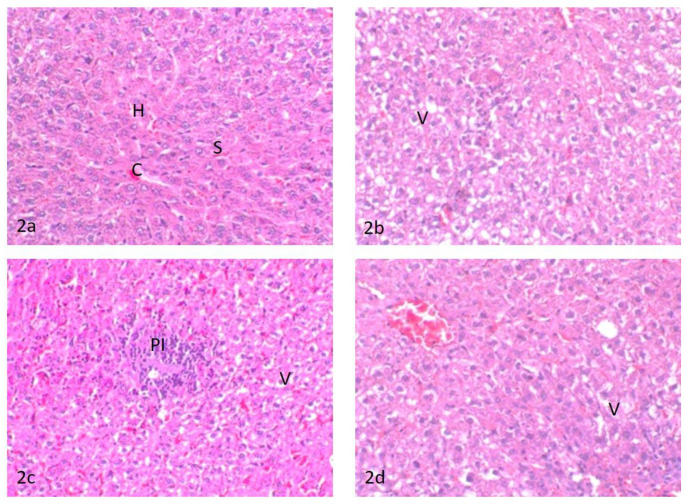
(**2a**) Liver of control untreated rats, central vein (C), sinusoid (S), and hepatocytes (H); (**2b**) liver of rats treated with water contaminated with dimethoate after remediation with TiO_2_(s)/H_2_O_2_/UV, hepatocellular vacuolar degeneration (V)’; (**2c**) liver of rats treated with water contaminated with methomyl after remediation with TiO_2_(s)/H_2_O_2_/UV, (PI) portal mononuclear cell infiltration and hepatocellular vacuolar degeneration (V); (**2d**) liver of rats treated with water containing TiO_2_(s)/H_2_O_2_ without any insecticides, hepatocellular vacuolar degeneration (V).

The degradation of the tested insecticides by TiO_2_(c)/H_2_O_2_/UV and TiO_2_(s)/H_2_O_2_/UV as a function of the irradiation time is shown in Figure 1 and Figure 2. The irradiation under the TiO_2_(s)/H_2_O_2_/UV system exhibited the highest degradation rate of the tested insecticides, followed by TiO_2_(c)/H_2_O_2_/UV. Complete degradation of the tested insecticides (100%) was achieved using the TiO_2_(s)/H_2_O_2_/UV system after 320 min of irradiation. The degradation rate constants and half-lives of the tested insecticides under the tested treatments are shown in Table 1. The half-life values of the tested insecticides under TiO_2_(c)/H_2_O_2_/UV were 43.86 and 36.28 for dimethoate and methomyl, respectively. Whereas, under the TiO_2_(s)/H_2_O_2_/UV system, the half-life values of the tested insecticides were 27.72 and 19.52 min for dimethoate and methomyl, respectively. The degradation of the insecticides tested in the dark using the different systems was negligible compared with that in light conditions. The degradation percentages of the tested insecticides under dark conditions using the different systems ranged from 0.30 to 1.6%.

#### 3.2.1. Biochemical Parameters

The complete detoxification of the tested insecticides in water samples treated with H_2_O_2_/TiO_2_(nano)/UV was confirmed by measuring some biochemical parameters in treated rats (AChE, GPT, GOT, and GST) versus the control. The results demonstrated that no significant differences were observed in the biochemical parameters (cholinesterase, GPT, GOT, and GST levels) measured in rats administrated with water containing insecticides after remediation with H_2_O_2_/TiO_2_(s)/UV relative to control (Table 2). Moreover, the effect of H_2_O_2_/TiO_2_(s) without insecticides on the same biochemical parameters showed no significant differences relative to control.

#### 3.2.2. Histopathological Changes

The complete detoxification of dimethoate and methomyl in water treated with TiO_2_(s)/H_2_O_2_/UV was confirmed regarding the histopathological changes in the liver and kidneys of treated rats compared to the untreated control.

##### Liver

The liver of control rats showed the basic feature of a hepatic lobule with a centrally located central vein. The hepatocytes were arranged in cords separated from each other by small blood spaces called hepatic sinusoids; the portal area contained the bile duct, hepatic artery, and portal vein (Figure 2a). The liver of rats treated with water contaminated with dimethoate after remediation with TiO_2_(s)/H_2_O_2_/UV showed moderate hepatocellular cytoplasmic vacuolation (Figure 2b). Meanwhile, the liver of rats treated with water contaminated with methomyl after remediation with TiO_2_(s)/H_2_O_2_/UV showed a slight hepatocellular cytoplasmic vacuolation and focal accumulation of mononuclear inflammatory cell infiltration in the portal area (Figure 2c). Conversely, the liver of rats treated with water containing TiO_2_(s)/H_2_O_2_ without any insecticides showed slight sinusoidal congestion and mild vacuolation in the cytoplasm of the hepatocytes (Figure 2d). A summary of histopathologic lesions in the liver of rats treated with water contaminated with methomyl and dimethoate after remediation with TiO_2_ (nano)/H_2_O_2_/UV is shown in Table S1 in the Supplementary Data.

##### Kidney

**Figure 3:**
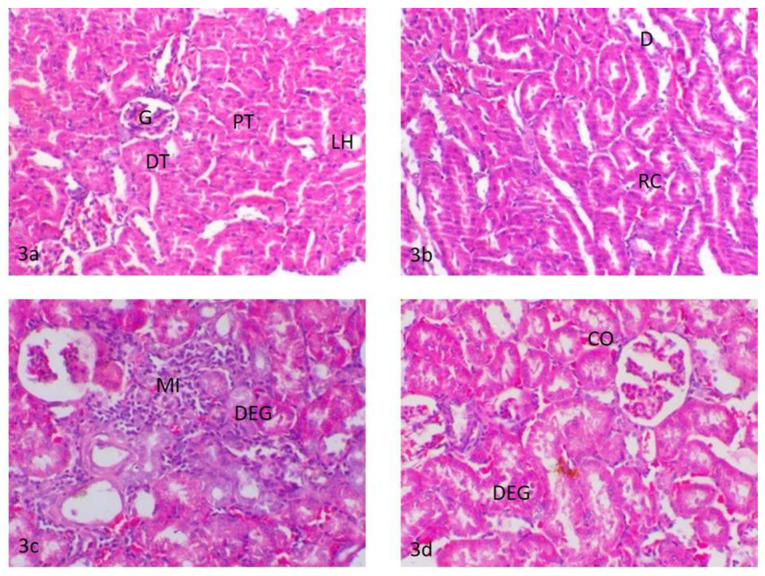
(**3a**) Kidneys of control rats, glomerulus (G), proximal convoluted tubules (PT), distal convoluted tubules (DT) and loop oh henle (LH); (**3b**) kidneys of rats treated with water contaminated with dimethoate after remediation with TiO_2_(s)/H_2_O_2_/UV, renal casts (RC) and tubular dilatation (D); (**3c**) kidneys of rats treated with water contaminated with methomyl after remediation with TiO_2_(s)/H_2_O_2_/UV, periglomerular and intertubular mononuclear cell infiltration (MI) and degeneration of the renal tubules (DEG); (**3d**) kidneys of rats treated with water containing TiO_2_(s)/H_2_O_2_ without any insecticides, degeneration of the renal tubules (DEG) and intertubular blood vessel congestion (CO).

The kidneys of control rats showed normal cortex and medulla. The cortex composed of a glomerular tuft of capillaries surrounded by Bowman’s capsule forming glomeruli were dispersed within the proximal and distal convoluted tubules. The medulla was mainly composed of collecting ducts and loop of Henle (Figure 3a). The kidneys of rats treated with water contaminated with dimethoate after remediation with TiO_2_(s)/H_2_O_2_/UV showed a similar histologic structure as that of the control group except for slight changes in the form of the renal tubule epithelial cell swelling and a few proteinaceous casts in the lumen of the renal tubules (Figure 3b). The kidneys of rats treated with water contaminated with methomyl after remediation with TiO_2_(s)/H_2_O_2_/UV showed focal glomerulonephritis with a focal accumulation of mononuclear inflammatory cells around the glomeruli and in between the degenerated renal tubules as well as slight tubular dilatation (Figure 3c). The kidneys of rats treated with water containing TiO_2_(s)/H_2_O_2_ (without dimethoate or methomyl) showed mild degeneration of the epithelial lining of renal tubules and slight congestion of the intertubular blood vessels (Figure 3d). A summary of histopathologic lesions in the kidney of rats treated with water contaminated with methomyl and dimethoate after remediation with TiO_2_ (nano)/H_2_O_2_/UV is shown in Table S1 in the Supplementary Data.

## 4. Discussion

The application of heterogeneous photocatalytic water purification processes has gained considerable attention the last few years resulting from its effectiveness in degrading and mineralizing the recalcitrant organic compounds and also because of the possibility of using solar UV and the visible-light spectrum [5,6,7,16,17]. Recently, water treatments with a nanocatalyst have been considered as a potential treatment and are becoming increasingly available worldwide because of the effective production of nanomaterials with modest resources and less waste (i.e., reducing pollution). In the present study, the degradation rate of the tested insecticides was greatly enhanced by using a TiO_2_ nanocatalyst via an advanced oxidation process compared to the bulk process. The unique properties of the TiO_2_ nanocatalyst, such as its great surface, contribute to its high degradation ability. The enhancement in its degradation ability might be attributed to the greater surface area provided by the stabilized nanoparticles in the small particle size and reactivity, leading to a higher generation rate of hydroxyl radicals compared to that of normal particles, and subsequently, a higher degradation rate of organic pollutants [43,48]. The titanium dioxide nanocatalyst is very reactive because the active sites are located on the surface. Additionally, these nanocatalysts present low diffusional resistance and are easily accessible to the substrate molecules. Another important feature of nanomaterials is that their surface properties can be very different from those shown by their macroscopic or bulk counterparts [49]. In comparison with their microsized counterparts, the nanoparticles show higher catalytic activity because of their large specific surface, in which catalytically active sites are exposed [5,6,7,17,42,43].

The titanium dioxide catalyst, either in nano or bulk size, significantly degrade the tested insecticides when employed in the advanced oxidation process [5,50]. The degradation mechanism of the treated insecticides probably occurs because semiconductors such as TiO_2_, in the presence of light, generate valence band holes (h^+^_vb_) and conduction band electrons (e^−^_cb_)—Equation (4). Hydroxyl free radicals are then generated through water oxidation by the photogenerated valence band holes according to Equations (5) and (6). The generated hydroxyl-free radicals react rapidly and non-selectively with organic molecules such as methomyl and dimethoate, leading to the production of numerous oxidation intermediates and final mineralization products, Equation (7) or react directly with the holes—Equation (8). In the presence of air, other species might contribute to the dimethoate or methomyl oxidation, such as H_2_O_2_ or even superoxide radicals, which are produced by oxygen reduction through photogenerated conduction band electrons—Equation (9), or H_2_O_2_ cleavage by conduction band electrons as represented in Equation (10).

TiO_2_ + hυ → e^−^_cb_ + h^+^_vb_ (4)

TiO_2_ (h^+^) + H_2_O → TiO_2_ + •OH + H (5)

TiO_2_ (h^+^) + OH → TiO_2_ + •OH (6)

Insecticide + •OH → intermediates → H_2_O + CO_2_ (7)

R + h^+^→ degradation products →→ H_2_O + CO_2_ (8)

O_2_ + e^−^_cb_ → O^−·^_2_ (9)

H_2_O_2_ + e^−^ → OH^·^ + OH^−^ (10)

After the first hour of irradiation, the degradation rate of the treated insecticides became slower under all photochemical remediation systems. This might be attributed to the low concentration of the remaining insecticides (<20% of its initial concentration) that leads to a high delivery rate of H_2_O_2_, which corresponds to higher reagent concentration, and subsequently, an increased ability to compete with the tested insecticides to react with hydroxyl radicals as a scavenger—Equation (11) [16]. Furthermore, the chloride and carbonate ions naturally present in water act as hydroxyl radical scavengers—Equations (12) and (13) [51]. All these reasons lead to the low degradation rate of the studied insecticide after one hour of irradiation.

OH + H_2_O_2_ → HO_2_^·^ + H_2_O (11)

Cl^−^ + ·OH → Cl^·^ + OH^−^ (12)

CO_3_^−2^ + ·OH → CO_3_^−·^ + OH^−^ (13)

Regarding the degradation of the tested insecticides under different oxidation processes in the presence of UV light, the contribution of direct photolysis (UV absorption) is nearly negligible and the radiation energy emitted by the lamp is mainly absorbed by the different catalysts to generate hydroxyl radicals [52]. The degradation rate of the tested insecticides by (TiO_2_(s)/H_2_O_2_ and TiO_2_(c)/H_2_O_2_) in the dark was quite low, and the induced degradation is probably due to the chemical hydrolysis of the tested insecticides since the tested catalyst is not active in the dark [16]. In this study, the results for the methomyl degradation agree with those previously reported [53,54,55], in which the total disappearance of methomyl and other carbamate insecticides completely mineralized was observed in AOPs using regular and nano-sized photocatalysts under UV light. Additionally, the results regarding the photocatalytic degradation of dimethoate agree with the results previously reported [56], in which the total disappearance of dimethoate and more than 90% degradation of other organophosphorus insecticides were observed in the AOPs system using regular and nano-sized photocatalysts under UV radiation.

The theoretical half-lives of the examined insecticides do not match with the experimental results, probably because of the experimental uncertainties in irradiation and also the analytical procedures, which are often underestimated [57,58]. The total detoxification of pesticide-contaminated water after remediation considers a limiting factor in the evaluation of the efficacy of any remediation technologies. The present results showed that the treated water samples after remediation had no significant effect on the activity of the measured biochemical parameters relative to control, which implies that the tested insecticides were completely detoxified [59]. In this context, the histopathological changes provide a rapid method to detect the effects of irritants in various tissues and organs [60]. The liver is considered the main organ for detoxification, which suffers serious morphological alterations as a result of exposure to pesticides [61,62]. Alterations in the markers of the liver could reflect the prior exposure to a series of environmental stressors. A toxicity assessment was performed to assess the efficacy of different studied remediations in the removal of dimethoate and methomyl from freshwater regarding the histopathological changes in rats. The exposure to Dimethoate and methomyl could result in a series of histopathological alterations in the liver and kidneys [63]. The degenerative changes in the liver and nephritic damage associated with dimethoate and methomyl were attributed to their induced oxidative stress, lipid peroxidation, and the resultant free radical accumulation [64]. In the present study, there were non-significant but only mild alterations in the liver and kidneys of rats treated with the synthesized nanoparticles. The rats treated with dimethoate or methomyl after remediation showed moderate hepatocellular cytoplasmic vacuolation and a focal inflammatory reaction in the portal area of the liver and focal glomerulonephritis, degenerated renal tubules, and the presence of a few proteinaceous casts in the lumen of the renal tubules. These changes are considered mild retrogressive degenerative changes, indicating a non-harmful effect of dimethoate or methomyl after remediation in comparison with the toxic effect of the exposure to dimethoate and methomyl in the liver and kidneys without remediation [65]. Furthermore, these effects are considered an adaptive physiological response attempting to limit cell damage that could occur by the presence of toxic insecticides or their metabolites. The mild inflammatory reaction observed in the liver and kidneys of rats treated with water contaminated with methomyl after remediation with TiO_2_(s)/H_2_O_2_/UV might be a defensive response of the tissues either to the oxidative stress-induced injury imposed by methomyl or to bacterial infection since some reports linked the possible influence of pesticides on alteration in the gut microbiome and possibly immunosuppression effects [8,66,67], suggesting further future investigations. The obtained results indicated low toxicity of the TiO_2_ nanocatalyst on the liver and kidneys [68]. Revising the available literature, similar mild histopathological and degenerative changes were relevant in kidney, liver, and other organs following a single exposure and a similar duration of treatment with some chemical or pesticide [69,70,71]. It seems that the time between administration is important. In this respect, Metalaxyl induced several histopathological alterations in the livers of rats after exposure to fungicide for 21 consecutive days [71]. These histopathological changes in the liver included cellular infiltration, condensation and fragmentation of nuclei, hydropic degeneration, and apoptosis in hepatocytes [71]. Similarly, mild degenerative changes were reported in the tubular epithelium together with cell swelling and severe interstitial mononuclear cell infiltration in the kidney following treatment with metalaxyl [71]. Moreover, Descotes et al., 1996 [70], reported focal basophilia of renal tubules in rats following daily intragastric treatment with cyclosporine A. Another study reported that a high dose (2 mg/kg) of cymoxanil in rats showed piecemeal necrosis in the liver combined with interstitial nephritis and tubular degeneration in the kidney [72]. In the same line, no obvious changes were recorded in the brain of rats following treatment with a low dose (0.5 mg/kg) of malathion for 21 consecutive days [69].

These results verify the safety of TiO_2_(s)/H_2_O_2_/UV use in water remediation for human health [73]. Furthermore, the nanocatalyst itself did not induce any significant toxicity on treated rats regarding biochemical and histological alterations in the treated rats. Although nanomaterials present seemingly limitless possibilities, they still bring with them new challenges to understand, predict, and manage the potential safety and health risks to humans. Even though the toxicological experiment described in this study showed that nanomaterials can be safe, it should be borne in mind that the implications of these engineered nanoparticles (NPs) are uncertain because they have different particle properties [74]. It is very important to note that the toxicity test in this study is insufficient for assessment of the safety for humans, as the exposure time was limited, the exposure conditions do not account for interspecies variability, and the measured endpoints were limited. In addition, the present study demonstrates the disappearance of the parent compounds, but not mineralization. Clearly, complete detoxification of breakdown products was confirmed, but the presence of breakdown products was not investigated. Therefore, further studies are required for the identification of possible breakdown and to determine the key physical and chemical characteristics of nanoparticles determining their hazard potential.

## 5. Conclusions

Altogether, the used AOPs were promising within the complete degradation and detoxification of the tested insecticides in contaminated water, especially by using the titanium dioxide nanocatalyst. The detoxification test (histopathological and biochemical tests), by exposing the treated water to a sensitive target, is considered an expressive measure for the complete removal of pesticide toxicity from the treated water compared to relying on the total degradation pesticides. Taking this into account, the pesticide can be completely destroyed, but the products of its breakdown can be more toxic than the compound itself, explaining the importance of the assessment of complete detoxification. Clearly, further efforts are needed for the identification of possible breakdown products, and future studies are suggested to verify the safety of the nanomaterials.

## Figures and Tables

**Table 1:** Degradation rate and half-life values of the tested insecticides under different irradiation systems.

Treatments	Degradation Rate Constant (day^−1^)	Half-Life (t_1/2_) (day)	R^2^
Methomyl			
H_2_O_2_/TiO_2_(c)/UV	0.0191	36.28	0.98
H_2_O_2_/TiO_2_(s)/UV	0.0355	19.52	0.95
Dimethoate			
H_2_O_2_/TiO_2_(c)/UV	0.0158	43.86	0.96
H_2_O_2_/TiO_2_(s)/UV	0.0250	27.72	0.94

**Table 2:** Effect of TiO_2_(s)/H_2_O_2_/UV with and without insecticides on the activity of some biochemical parameters in rats.

Treatment	AChE	GPT	GOT	GST
M + H_2_O_2_/TiO_2_(s)/UV	0.912 × 10^−1^ ± 0.001 ^a^	51.89 ± 0.29 ^a^	0.0113 ± 0.0011 ^a^	19.010 ± 0.62 ^a^
D + H_2_O_2_/TiO_2_(s)/UV	0.912 × 10^−1^ ± 0.001 ^a^	51.78 ± 0.93 ^a^	0.0114 ± 0.0012 ^a^	19.040 ± 0.61 ^a^
H_2_O_2_/TiO_2_(s)/UV	0.912 × 10^−1^ ± 0.001 ^a^	51.78 ± 0.93 ^a^	0.0114 ± 0.0011 ^a^	19.040 ± 0.61 ^a^
Control	0.911 × 10^−1^ ± 0.001 ^a^	51.4 ± 0.013 ^a^	0.0113 ± 0.008 ^a^	19.040 ± 0.68 ^a^

M = methomyl, D = dimethoate, ^a^ mean there is no significant difference between the means.

## Data Availability

The data that support the findings of this study are available on request from the corresponding author.

## Supplementary Materials

The following are available online at https://www.mdpi.com/article/10.3390/ijerph18179278/s1, Figure S1: SEM image of the synthesized (a) commercial (b) titanium dioxide, Figure S2: TEM image of the synthesized (a) commercial (b) titanium dioxide. Figure S3: Diffuse reflectance UV-Vis spectra of synthesized TiO_2_ (a) and commercial TiO_2_ (b). Figure S4: XRD patterns of synthesized (A) and commercial (B) TiO_2_. Table S1: Summary of histopathologic lesions in the liver and kidneys of rats treated with water contaminated with methomyl and dimethoate after remediation with TiO_2_ (nano)/H2O_2_/UV.
