# Supplementary material for: Photocatalytic Detoxification of Some Insecticides in Aqueous Media Using TiO2 Nanocatalyst
Source: Int J Environ Res Public Health. 2021 Sep 2;18(17):9278. doi: 10.3390/ijerph18179278 (PMC8431621; doi:10.3390/ijerph18179278)
Supplement: Supplementary file 1 [file ijerph-18-09278-s001.zip › ijerph-1318039-supplementary.pdf]

International Journal of
*Environmental Research and Public Health*

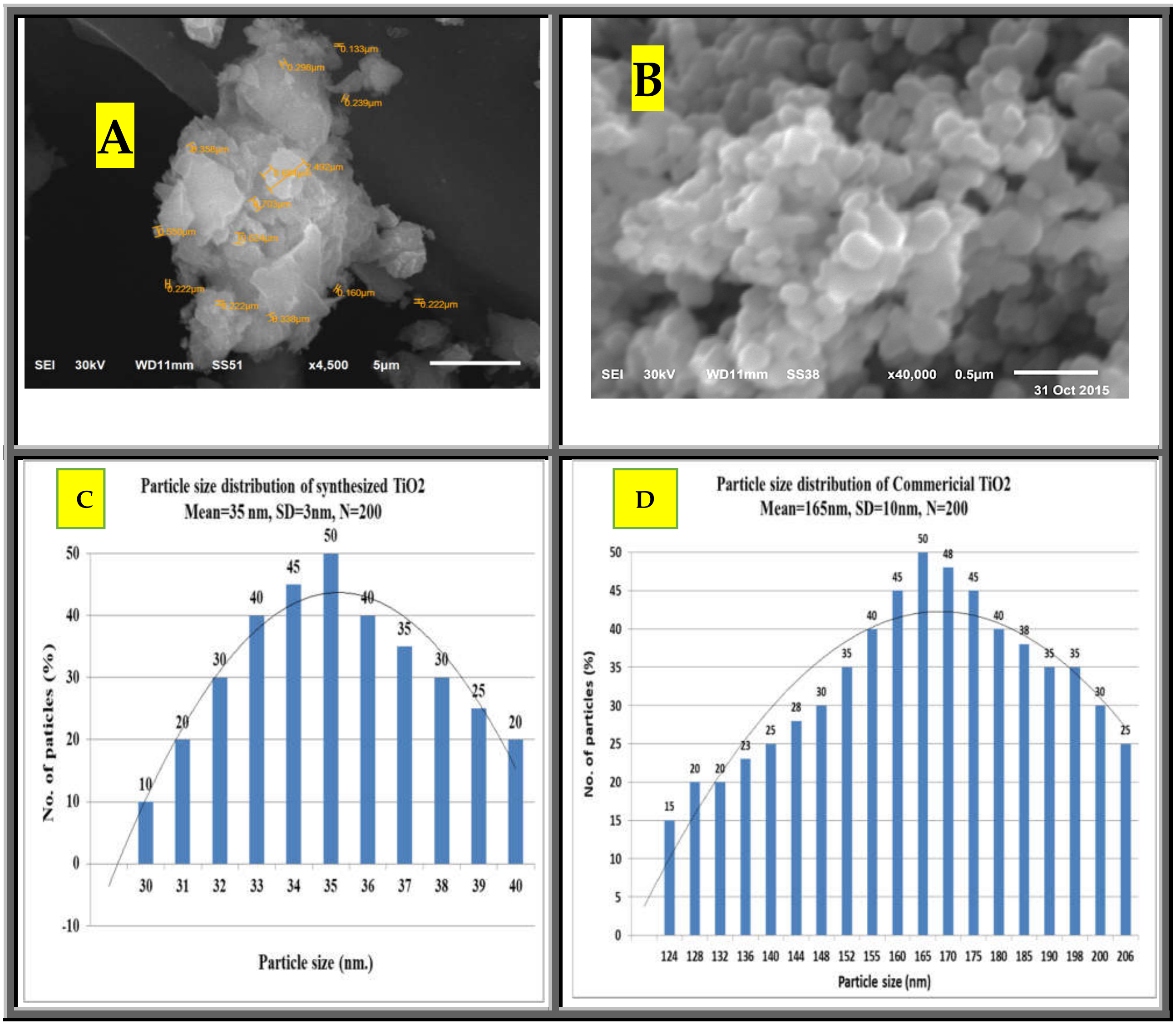


**Figure S1.** SEM image and particles size disrubtion of the synthesized (**A**,**C**) commercial (**B**,**D**) titanium dioxide. d = mean diameter, SD = standard deviation and N = number of particles counted.

International Journal of
*Environmental Research and Public Health*

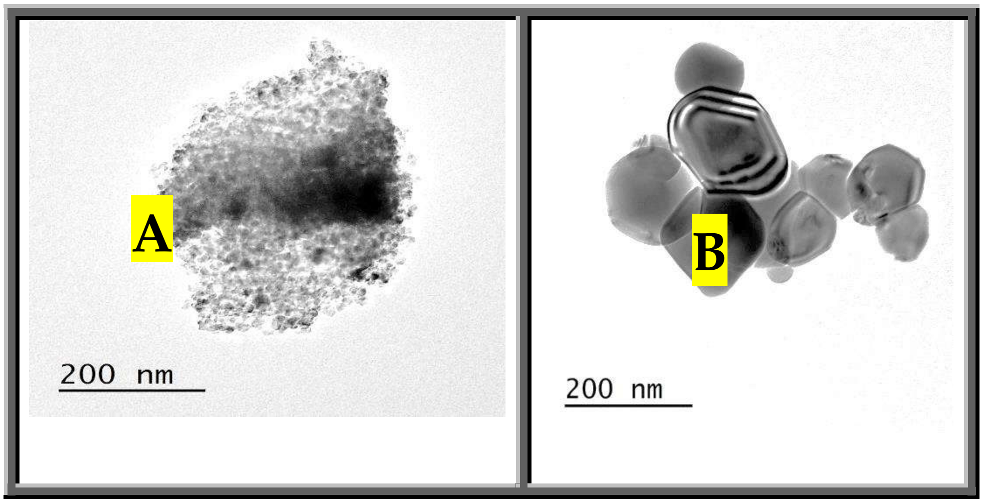


**Figure S2.** TEM image of the synthesized (a) commercial (b) titanium dioxide.

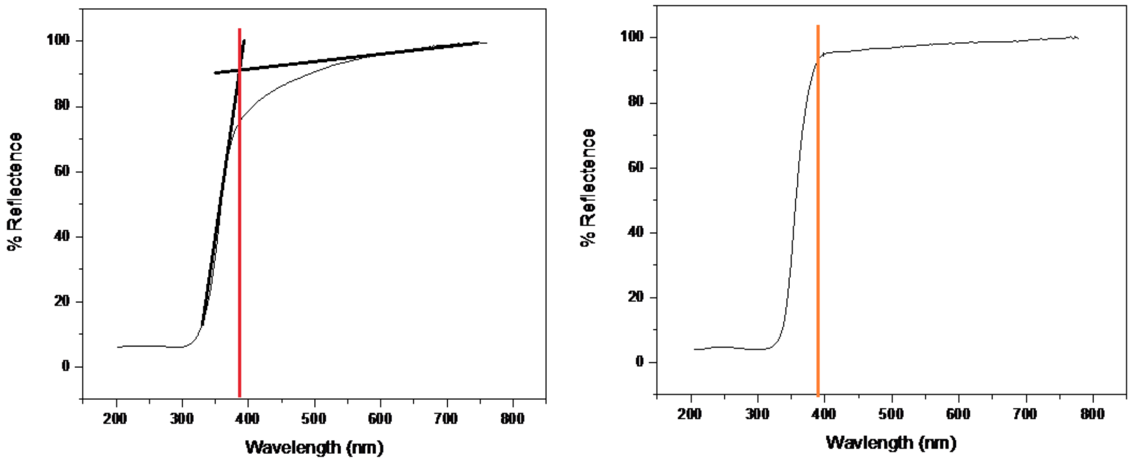


(a) (b)

**Figure S3.** Diffuse reflectance UV-Vis spectra of synthesized $TiO_2$ (**A**) and commercial $TiO_2$ (**B**).

International Journal of
*Environmental Research and Public Health*

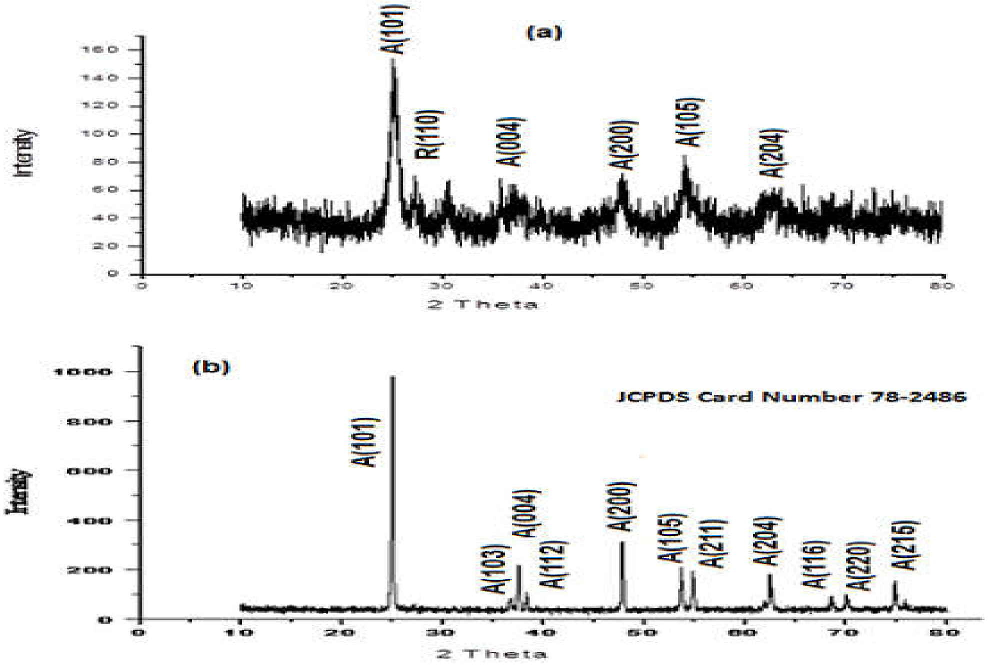


**Figure S4.** XRD patterns of synthesized (**a**) and commercial (**b**) $TiO_2$

**Table S1.** Summary of histopathologic lesions in the liver and kidneys of rats treated with water contaminated with methomyl and dimethoate after remediation with $TiO_2$ (nano)/$H_2O_2$/UV.

| Organ | | liver | | | kidney | | | | |
|---|---|---|---|---|---|---|---|---|---|
| lesions | | vacuolation | inflam | congestion | Tubular deg | Renal cast | inflam | Tubular dilat | congestion |
| Control | • 1 | - | + | - | + | - | - | - | - |
| | • 2 | - | - | - | - | - | - | - | - |
| | • 3 | + | - | + | - | - | - | - | - |
| | • 4 | - | - | - | - | - | - | - | - |
| | • 5 | - | - | - | - | - | + | - | + |
| | • 6 | - | - | + | - | - | - | - | - |
| dimethoate | • 1 | ++ | + | - | + | + | - | - | - |
| | • 2 | ++ | - | + | - | + | - | - | - |
| | • 3 | ++ | - | + | + | + | - | + | - |
| | • 4 | + | + | - | + | - | - | - | - |
| | • 5 | + | - | - | + | + | - | + | - |
| | • 6 | ++ | - | - | + | - | + | - | - |
| Methomyl | • 1 | + | - | + | + | - | + | + | - |
| | • 2 | + | + | - | + | - | ++ | + | + |
| | • 3 | + | + | - | - | + | + | + | + |
| | • 4 | ++ | + | + | + | - | + | + | - |
| | • 5 | + | + | - | - | - | - | - | - |
| | • 6 | + | + | - | + | - | + | - | - |
| TiO2 (nano) /(nano)/H2O2/UV | • 1 | + | - | + | + | - | - | - | + |
| | • 2 | - | + | + | - | - | + | - | + |
| | • 3 | + | - | + | - | - | - | - | - |
| | • 4 | + | - | + | + | - | - | - | + |
| | • 5 | + | - | - | + | - | - | - | + |
| | • 6 | ++ | - | + | + | - | - | - | + |

deg: degeneration; inflam: inflammation; dil: dilataion; -Normal; + Mild; ++ Moderate; +++ Severe
